# Complete Genome Sequence of the Yogurt Isolate *Lactobacillus delbrueckii* subsp. *bulgaricus* ACA-DC 87

**DOI:** 10.1128/genomeA.00868-17

**Published:** 2017-08-24

**Authors:** Voula Alexandraki, Maria Kazou, Bruno Pot, Effie Tsakalidou, Konstantinos Papadimitriou

**Affiliations:** a Laboratory of Dairy Research, Department of Food Science and Human Nutrition, Agricultural University of Athens, Athens, Greece; b Research Group of Industrial Microbiology and Food Biotechnology (IMDO), Vrije Universiteit Brussel, Brussels, Belgium

## Abstract

*Lactobacillus delbrueckii* subsp. *bulgaricus* is widely used in the production of yogurt and cheese. In this study, we present the complete genome sequence of *L. delbrueckii* subsp. *bulgaricus* ACA-DC 87 isolated from traditional Greek yogurt. Whole-genome analysis may reveal desirable technological traits of the strain for dairy fermentations.

## GENOME ANNOUNCEMENT

*Lactobacillus delbrueckii* subsp. *bulgaricus* is among the most important starters employed in dairy fermentations and is used mainly in the production of yogurt in protocooperation with *Streptococcus thermophilus* (1). *L. delbrueckii* subsp. *bulgaricus* belongs to the acidophilus complex, a group of lactobacilli which further includes *Lactobacillus acidophilus*, *Lactobacillus helveticus*, *Lactobacillus johnsonii*, and *Lactobacillus gasseri* (2). Here, we report the genome sequencing and the genomic features of *L. delbrueckii* subsp. *bulgaricus* ACA-DC 87, which was isolated from traditional Greek yogurt (3, 4).

The genome was sequenced at the Beijing Genomics Institute (BGI Co., Ltd., Hong Kong). A total of 17,362,540 paired-end reads (500-bp, 2,000-bp, and 6,000-bp libraries) were generated using the Illumina HiSeq 2000 platform with >100-fold sequence coverage. After filtering, the reads were assembled with SOAPdenovo version 1.05, resulting in one circular chromosome (5). The accuracy of the assembly was evaluated through whole-genome alignment of the ACA-DC 87 genome against a reference genome, namely, that of *L. delbrueckii* subsp. *bulgaricus* ATCC 11842 (6), using ProgressiveMauve (7). The ACA-DC 87 genome sequence was analyzed using 3 gene prediction programs, i.e., Prodigal (8), MetaGeneAnnotator (9), and FGENESB (10). Additionally, RAST version 2.0 was used both in genome annotation and prediction of rRNA and tRNA genes (11). The GenePRIMP pipeline was also employed for the detection of annotation anomalies, including putative pseudogenes (12). All the results obtained were manually curated. Further bioinformatics analysis focused on the identification of genomic islands (GIs) with IslandViewer 4 (13), clustered regularly interspaced short palindromic repeats (CRISPRs) with CRISPRFinder (14), and Pfam domain-containing proteins with Pfam database (15), while the WebMGA server was used for clusters of orthologous groups (COG) functional annotation (16).

The chromosome of ACA-DC 87 comprises 1,856,003 bp with a G+C content of 49.8%. A total of 1,993 genes were identified in the genome sequence, including 1,644 protein-coding genes and 229 potential pseudogenes. The genome also contains 93 tRNA genes and 9 complete rRNA operons. Twelve genomic islands (GIs) with a total of 196 genes were predicted in the genome of ACA-DC 87. These genes may have been acquired through horizontal gene transfer and could be related to technological features. Several of these genes encode CRISPR-associated proteins, subunits of restriction-modification systems, and proteins implicated in exopolysaccharide biosynthesis. The genome also carries one confirmed CRISPR array with a size of 761 bp, containing 11 spacers. Furthermore, 1,322 proteins with Pfam domains were identified. The COG annotation results revealed that approximately 87% of the protein-coding genes (1,284 proteins) were assigned to at least one functional category. The higher number of proteins were allocated to the category of translation, ribosomal structure and biogenesis (J: 8.5%), followed by the categories of amino acid transport and metabolism (E: 7.8%) and replication, recombination, and repair (L: 7.4%). Further analysis of the ACA-DC 87 genome sequence may decipher the technological potential of the strain toward its application in the production of fermented dairy products.

### Accession number(s).

The genome sequence of *L. delbrueckii* subsp. *bulgaricus* ACA-DC 87 was deposited at the European Nucleotide Archive under the accession number LT899687.
